# Supplementary material for: Resistance Mechanism to Metsulfuron-Methyl in Polypogon fugax
Source: Plants (Basel). 2021 Jun 28;10(7):1309. doi: 10.3390/plants10071309 (PMC8308939; doi:10.3390/plants10071309)
Supplement: Supplementary file 1 [file plants-10-01309-s001.zip › plants-1268013-supplementary/Supplemental files/Supplementary Figure S1.pdf]

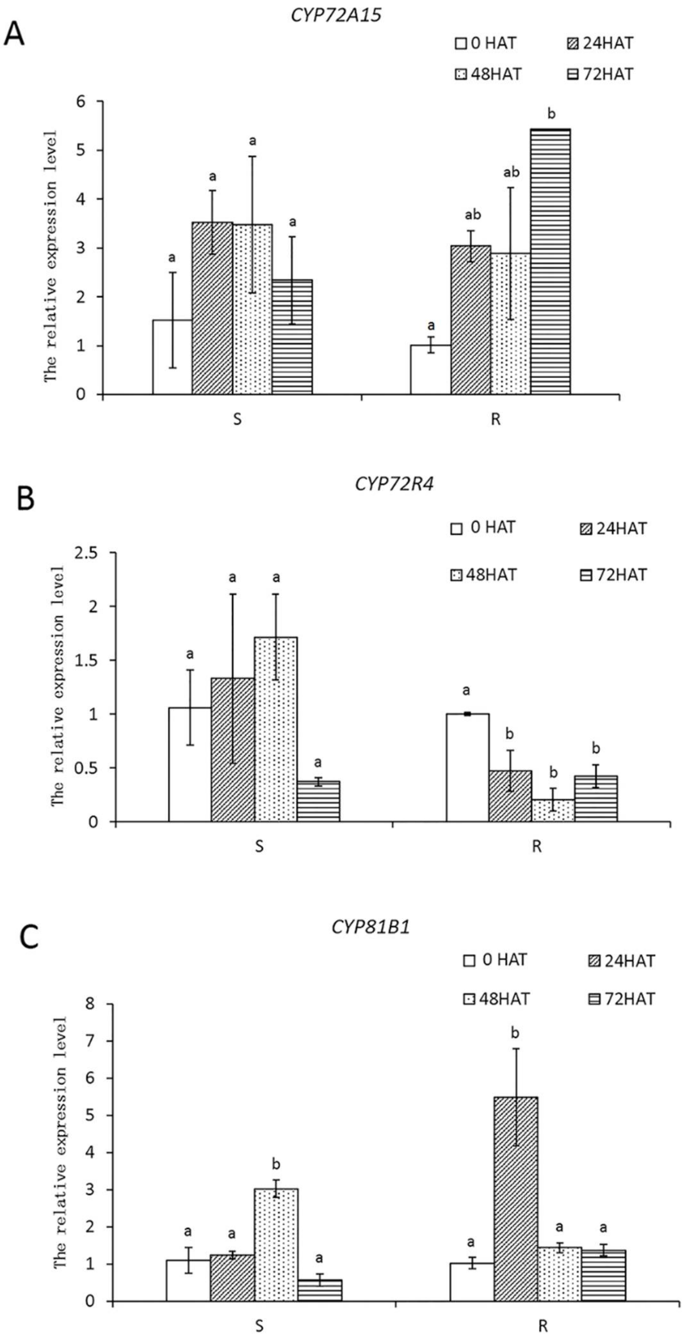

Figure S1. The relative expression of three *CYP* genes in R and S with metsulfuron-methyl treatment. The relative expression level of each gene (A. *CYP72A15*; B. *CYP72R4*; C. *CYP81B1*) were measured by RT-PCR. The 0 HAT AS and AR sample was considered as 1. Bars are mean ± standard error (n = 3). Values in a column followed by the same letter are not different according to Fisher's least significant differences test ($P \leqslant 0.05$).
